# Supplementary material for: Splice-Junction-Based Mapping of Alternative Isoforms in the Human Proteome
Source: Cell Rep. Author manuscript; Available in PMC 2020 Jan 15. (PMC6961840; doi:10.1016/j.celrep.2019.11.026)
Supplement: 2 [file NIHMS1546469-supplement-2.zip › DF1/PXD010154/Prostate/Prostate_12_ILVBL_LNSDIFWKPQEAVQGTVCDFRLSYGR.pdf]

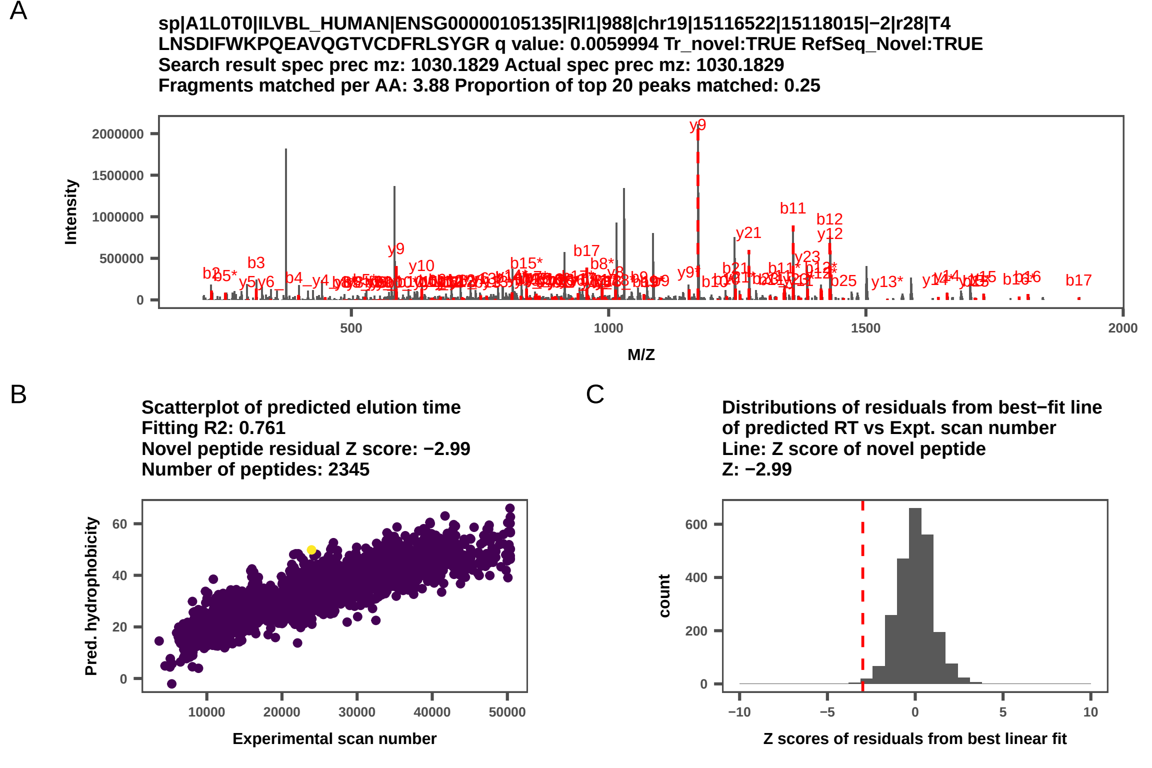
A
sp|A1L0T0|ILVBL_HUMAN|ENSG00000105135|RI1|988|chr19|15116522|15118015|−2|r28|T4
LNSDIFWKPQEAVQGTVCDFRLSYGR q value: 0.0059994 Tr_novel:TRUE RefSeq_Novel:TRUE
Search result spec prec mz: 1030.1829 Actual spec prec mz: 1030.1829
Fragments matched per AA: 3.88 Proportion of top 20 peaks matched: 0.25
Intensity
2000000
1500000
1000000
500000
0
M/Z
500
1000
1500
2000
y9
b11
b12
y12
y21
y23
b17
b8*
y10
b15*
b3
y5
y6
b4
y9*
b21
b25
y13*
b17
B
Scatterplot of predicted elution time
Fitting R2: 0.761
Novel peptide residual Z score: −2.99
Number of peptides: 2345
Pred. hydrophobicity
60
40
20
0
Experimental scan number
10000
20000
30000
40000
50000
C
Distributions of residuals from best−fit line
of predicted RT vs Expt. scan number
Line: Z score of novel peptide
Z: −2.99
count
600
400
200
0
Z scores of residuals from best linear fit
−10
−5
0
5
10
